# Addressing the overlooked frontier in AMR research and surveillance

**DOI:** 10.3389/fpubh.2025.1625515

**Published:** 2025-09-02

**Authors:** Rishu Thakur, Hena Dhar, Teresa M. Wozniak, Supriya Mathew

**Affiliations:** ^1^Menzies School of Health Research, Charles Darwin University, Alice Springs, NT, Australia; ^2^School of Biotechnology, Faculty of Applied Sciences and Biotechnology, Shoolini University, Solan, India; ^3^Australian e-Health Research Centre, CSIRO, Brisbane, QLD, Australia

**Keywords:** antimicrobial use, climate change, environment surveillance, multi-disciplinary approach, metagenomics

## Introduction

**Figure 1:**
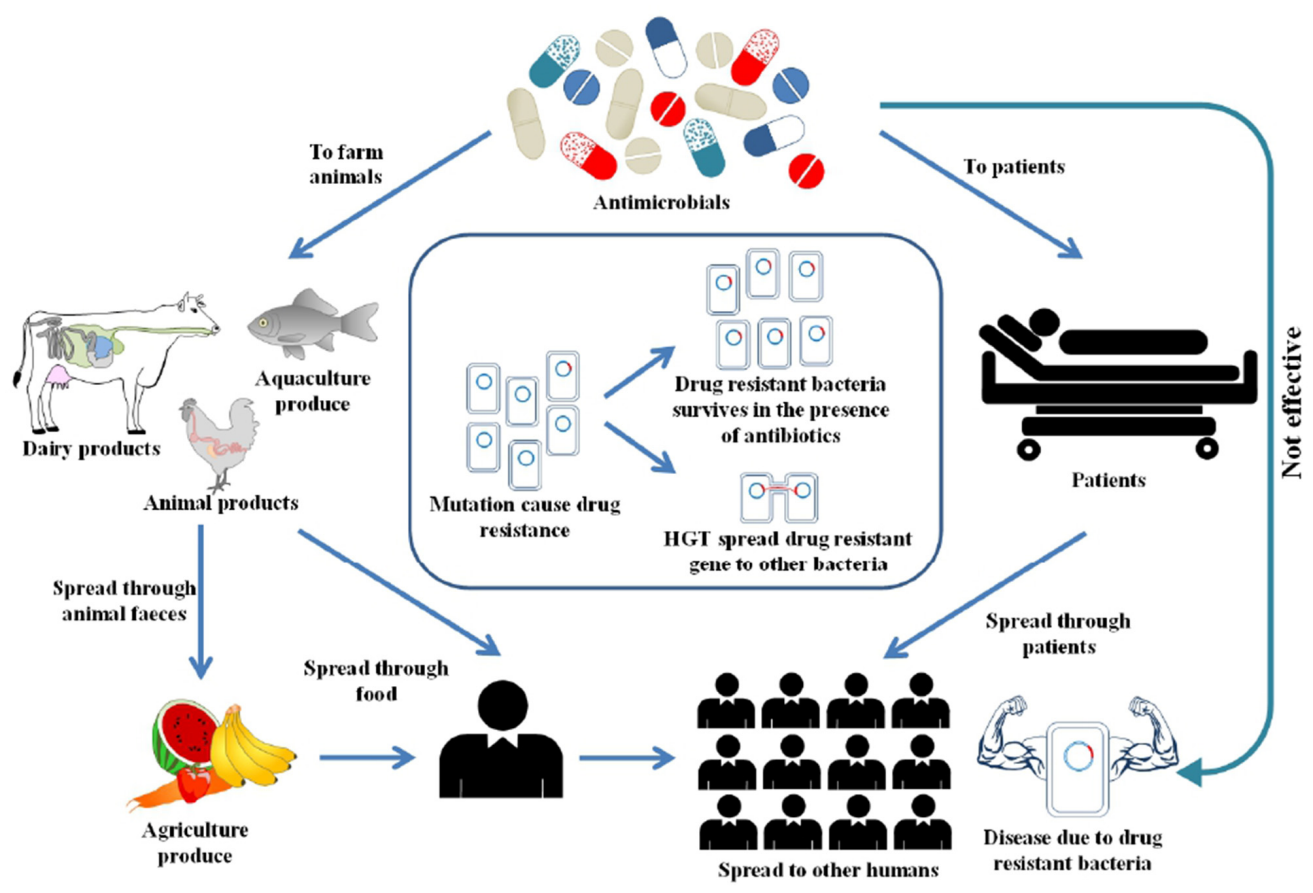
AMR emergence and propagation in the environment.

Antimicrobial Resistance (AMR) has been declared as one of the top ten global public health threats (1). There were an estimated 4.95 million deaths globally due to AMR in 2019, in which bacterial AMR alone was directly responsible for killing up to 1.27 million people (2). A World Bank report projected that a high AMR impact scenario could lead to a 3.8% decline in global annual GDP by 2050 (3). Humans are exposed to AMR through interconnected pathways, including healthcare, agriculture, animals and environment (4). Figure 1 illustrates the interlinked factors that drive AMR in the environment. However, the reality is more complex than depicted in the figure. AMR predominantly occurs from overuse of antimicrobials, that creates selective pressure, leading to the development of resistance against antimicrobials (5). Recent COVID-19 pandemic has significantly increased the use of antibiotics (6). This rise was influenced by several factors, including uncertainty in early diagnosis of COVID, limited treatment guidelines, concern over secondary bacterial infections, and overwhelmed healthcare systems (7). Besides the overuse of antibiotics, other factors such as socio-cultural and economic factors also influence the AMR spread (8). Vulnerable groups with pre-existing health conditions and high burden of diseases are most susceptible to AMR. For instance, remote Indigenous communities in Australia, who experience a high disease burden recorded high rates of azithromycin resistance, methicillin resistant *Staphylococcus aureus* and gram-negative resistance (9–11). Behavioral factors such as unnecessary antibiotic use, over the counter access to antibiotics without a prescription in low and middle-income countries (LMICs) and inappropriate disposal of antibiotics, can also affect the spread of AMR (12).

Overuse of antimicrobials is not exclusive to humans as large quantities of antimicrobials are use in food-producing animals (13). In healthcare settings, AMR surveillance is mainly used to guide immediate actions such as selecting the right antimicrobials or changing how antimicrobials are used (14). However, for environmental AMR, it is harder to link any results directly to immediate actions. One of the main reasons is that antimicrobial resistance genes (ARGs) naturally exist in the environment, such as in soil even with no known anthropogenic activity (15). The extent to which anthropogenic activities, such as the release of antimicrobials into the environment influences the naturally occurring AMR in environmental settings is largely unknown. There is no baseline data for AMR in environmental settings where antimicrobial residues accumulate through runoff. Next, how ARGs and antibiotic residues changes over time due to anthropogenic activities is largely unexplored. Estimating the level of AMR in these environments is difficult without sufficient baseline data that serve as a proper reference point for effective monitoring and evaluation of interventions.

## AMR in the face of climate change

According to the Lancet Countdown on Health and Climate Change, climate change has gradually increased disease risks which is expected to have significant impacts on the emergence and severity of AMR (16). Temperature variability and extreme weather events, such as heatwaves are increasing in frequency (17). Warmer temperatures have led to the emergence of climate-sensitive infections and are also associated with accelerating bacterial reproduction, which enhances the possibilities of horizontal gene transfer (18, 19). Elevated temperatures also influence heavy metal concentrations in the environment and their uptake by bacteria which can contribute to the proliferation of AMR (20). Climate uncertainties increase farmers reliance on antimicrobials as a defense against disease outbreaks and reduced crop yields (21). Other climate-mediated consequences, such as droughts exacerbate water and food insecurity, can lead to weakened immune systems, malnutrition, and increased susceptibility to infections (22). Floods also increase the risks of displacing populations and poor sanitation, which make ideal circumstances for infection spread (23). These challenges are further worsened by poor living conditions, overcrowded spaces and substandard housing, limited access to clean water and healthcare in low resource settings. Thus climate change is an underappreciated but critical driver of AMR, and hence, public health strategies must account for these emerging risks.

## Global efforts in AMR surveillance

The World Health Assembly initiated global action plans to tackle AMR, which vary for LMICs and high-income countries (HICs) (24). In LMICs, the focus is on issues like poor regulatory enforcement, and the unrestricted use of antibiotics; while in HICs, such as the European Union, action plans aim to strengthen AMR knowledge through surveillance, improve infection control and raise public awareness. Nevertheless, antibiotic usage is well monitored in healthcare settings among HICs; environmental settings need to be monitored to get a clear picture of AMR occurrence and propagation.

The World Health Organization (WHO) has made global efforts to enhance the AMR data collection through Global Antimicrobial Resistance and Use Surveillance System (GLASS) (25). GLASS collaborates with many regional AMR networks such as the Central Asian and European Surveillance of Antimicrobial Resistance (CAESAR), the European Antimicrobial Resistance Surveillance Network (EARS-Net), the Latin American Network for Antimicrobial Resistance Surveillance (ReLAVRA), and the Western Pacific Regional Antimicrobial Consumption Surveillance System (WPRACSS). Over 100 countries have participated in this surveillance till date. Despite the wealth of data collected through GLASS, some challenges continue in understanding and interpreting this data due to variable data quality across diverse regions and healthcare. GLASS report also indicated that many countries lack sufficient surveillance data on AMR, particularly LMICs. Another initiative, Global Antibiotic Research & Development Partnership (GARDP) provides data on AMR surveillance in LMICs (26). Recently, the G7 compliance report on AMR also emphasized on the AMR data collection across human, animal, and environmental health sectors in alignment with the “One Health” framework (27).

## Limited research on environmental AMR

Although global efforts have been made to improve AMR surveillance, research studies are more focused on clinical settings. Substantial efforts have been made toward monitoring AMR in clinical and veterinary settings (28). At present, many studies have investigated AMR in relation to humans, largely from the health perspective, followed by slightly fewer studies showing interest in the spread of AMR among animals and significantly less focusing on environmental factors contributing to AMR emergence. Given that the environment plays a significant role in the emergence and spread of AMR, it is important to develop more holistic approaches that comprise both healthcare and environmental. The absence of environmental surveillance is a critical gap in AMR research and limits the ability to provide evidence-based recommendations to policymakers. This imbalance emphasizes the pressing need for research aimed at elucidating the mechanisms underlying AMR development in environmental contexts.

One significant challenge is the lack of understanding of which environmental settings are most susceptible to AMR transmission. Such understanding is essential for targeted surveillance and development of interventions. High-risk environments where AMR transmission is most likely to occur need to be clearly defined. Another major obstacle is the research funding. Generally, clinical research, such as hospital infections related AMR receives more funding than research in environmental settings. Funding is generally allocated to antimicrobial stewardship in the healthcare sector. Funding for the One Health initiative is crucial to address the environmental AMR. A recent One Health initiative SAAFE AMR surveillance program funded by Australian Government recognizes the interconnection between people, animals, plants and their shared environments (29). More programs like this are needed to tackle this complex AMR issue.

## Future directions

### Local AMR surveillance through a multi-disciplinary approach

Global efforts are essential for AMR mitigation, but local solutions that can address the root causes of resistance in specific settings are also equally important. Several local factors can influence the AMR emergence. For instance, social determinants of health such as limited access to healthcare, poor housing and sanitation and geographical remoteness are well-known AMR contributors (8). Traditional farming practices, such as the use of fertilizers and economic pressure to increase production, could impact antimicrobial use. Such factors can be monitored through local surveillance that can provide insight into the micro-dynamics of AMR at ground levels. Local surveillance enables early detection of AMR reservoirs. This can be achieved by establishing local AMR monitoring networks among local/place-based researchers, health workers, farmers, community members and stakeholders which can help in identifying high-risk settings and prioritize local needs.

**Figure 2:**
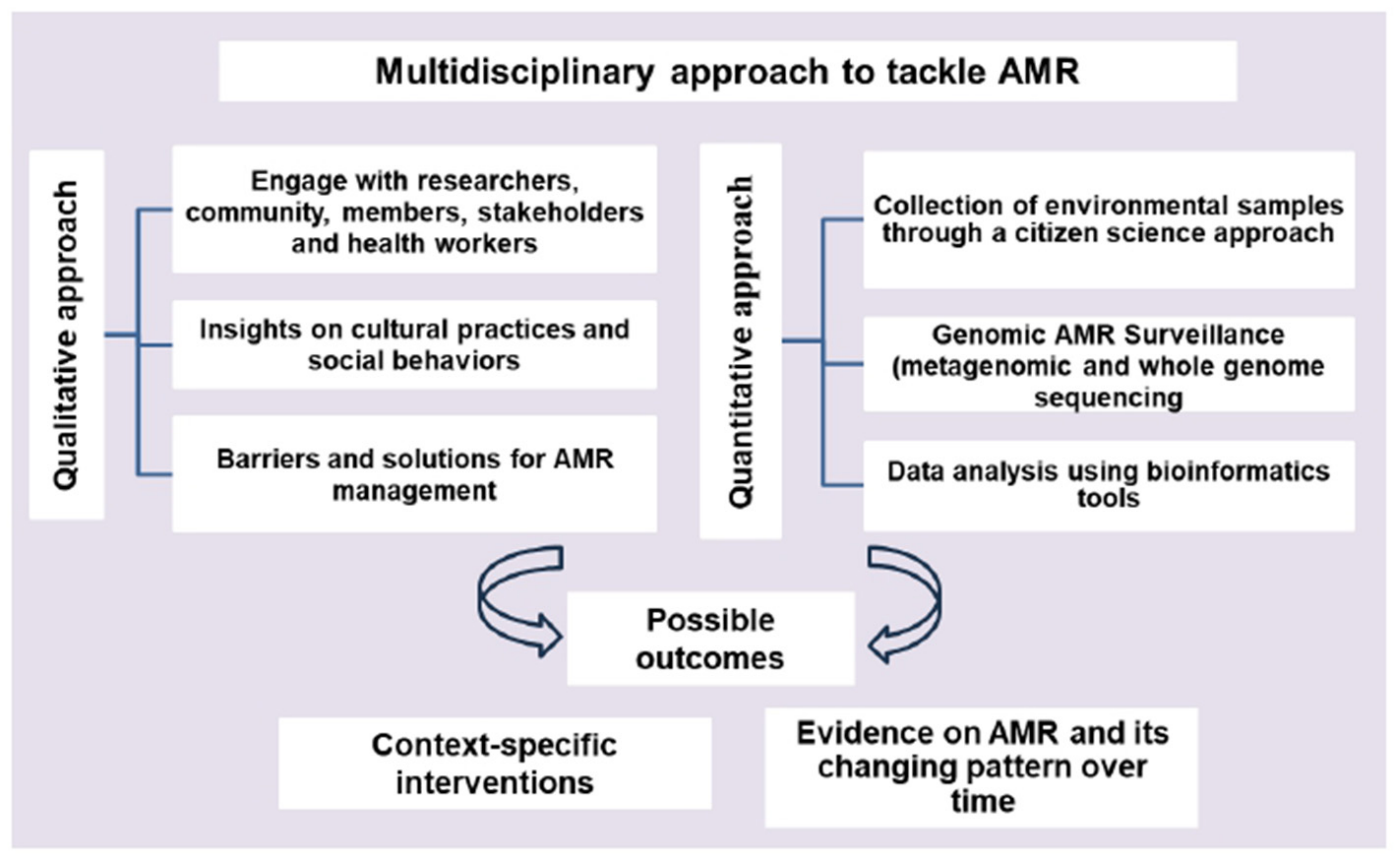
Multidisciplinary approach to monitor AMR emergence in the environment.

A combined qualitative and quantitative approach (Figure 2) can offer a comprehensive understanding, context and depth of the problem. Qualitative approaches can provide insights into how cultural practices and social behaviors influence the use of antimicrobials (30). Knowing the concerns of the communities and collecting environmental data through a citizen science approach can unlock many benefits, such as improving data quantity and quality, engaging communities and stakeholders and increasing awareness. Samples that citizen scientists can gather might come from a broader range of locations. Metagenomics including whole genome sequencing (WGS) can provide useful information about how resistance spreads within and between different reservoirs (31). Recently, many studies showed the potential of machine learning methods in predicting AMR with WGS methods (32). Using these techniques in local settings could facilitate rapid data collection and analysis of resistant genes and strains, with outcomes that support implementation of context specific interventions. However, the application of genomic approaches has been mostly restricted to research, and a lack of awareness, global co-ordination and political will to invest in using these technologies in active monitoring is a major obstacle that must be overcome.

## Key recommendations

- Stable funding is critical for long-term AMR monitoring. More funding should be allocated towards multi-disciplinary and One Health approaches.
- Continuous monitoring of antimicrobial use, and AMR awareness among the public and stakeholders could help in AMR surveillance and stewardship.
- Investing in scalable and locally informed AMR surveillance strategies is essential to achieve long-term AMR control at both the community and global levels.
- Capacity building among researchers, stakeholders, general practitioners, nurses and community members is needed.
- Parallel efforts should be carried out to reduce antimicrobial use and spread of infections in humans and animals.
